# An Explorative Note on Apraxia Tests

**DOI:** 10.3389/fneur.2018.00660

**Published:** 2018-08-08

**Authors:** Philipp Gulde, Katharina Leippold, Alan Armstrong, Sarah Kohl, Timo Grimmer, Janine Diehl-Schmid, Joachim Hermsdörfer

**Affiliations:** ^1^Chair of Human Movement Science, Department of Sport and Health Sciences, Technical University of Munich, Munich, Germany; ^2^Department of Psychiatry & Psychotherapy, Klinikum Rechts der Isar, Technical University of Munich, Munich, Germany

**Keywords:** apraxia, motor capacity, dementia, principal component analysis, imitation

## Abstract

Apraxia is stated independent of primary motor disorders. However, patient groups suffering from stroke or dementia can reveal motor impairments. In this study we examined the dependence of apraxia tests of imitation and pantomime on a latent motor component using a principal component analysis. With samples sizes of 11 patients suffering from dementia of the Alzheimer's type and 15 healthy control subjects, clear limitations concerning the validity of the results are given. Nevertheless, we could observe strong dependence of the three apraxia tests, especially the imitation of finger and hand gestures, on a latent motor component in this preliminary examination. We suggest confirmation by larger samples sizes and to control for the basic motor capacity when testing for signs of apraxia in such patient samples.

## Introduction

Apraxia describes the inability to perform actions with familiar tools in an “effective and purposeful manner” (1). Apraxia can also impair imitating actions, e.g., hand gestures, and pantomiming the use of objects (2–4). It is stated independent of “primary motor disorders” (4) and is denoted a “higher order disturbance of motor control” (3). A variety of studies revealed that the movement kinematics in apraxia are dependent on the mode of execution (5, 6). Since the frequency of apraxia in stroke patients with left brain damage is reported high with 28 to 51% (7, 8), a lot of research is focusing on stroke patients. However, apraxia is also prevalent in dementia patients, especially when suffering from dementia of the Alzheimer's type with estimates of 35 to 98% (9, 10). Dementia patients show impairments in fine and gross motor control (11, 12), even in the early form of mild cognitive impairment (13) and apart from extrapyramidal signs (14). The apraxia tests of imitating meaningless finger and hand gestures and pantomiming the use of everyday objects by Goldenberg (15) request upper-limb movements from patients that have, apart from the cognitive load of imitating and pantomiming, relevant demands on motor capacity. In stroke patients this can be partially controlled for by using the hand ipsilesional to the lesion, while in dementia the motor impairments usually impact both upper limbs. In this note we examined the aforementioned tests in a small sample of dementia patients to estimate their dependence on motor capacity.

## Methods

**Figure 1:**
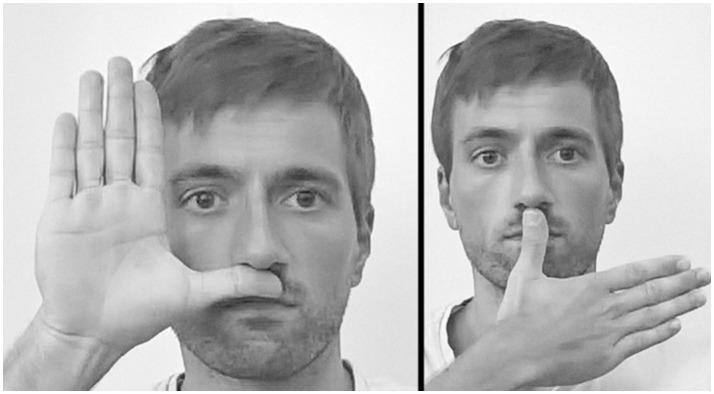
Imitation of meaningless hand gestures. Two examples of the 10 assessed gestures.

**Figure 2:**
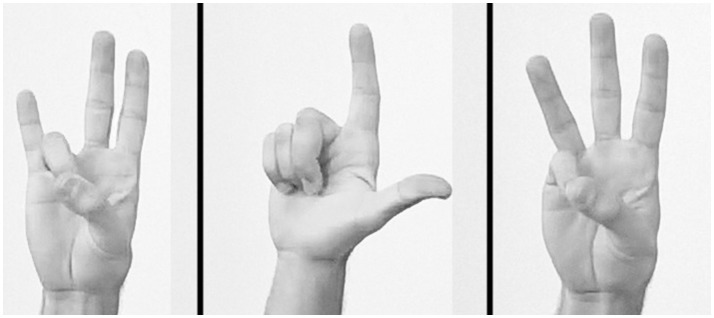
Imitation of meaningless finger gestures. Three examples of the 10 assessed gestures.

**Figure 3:**
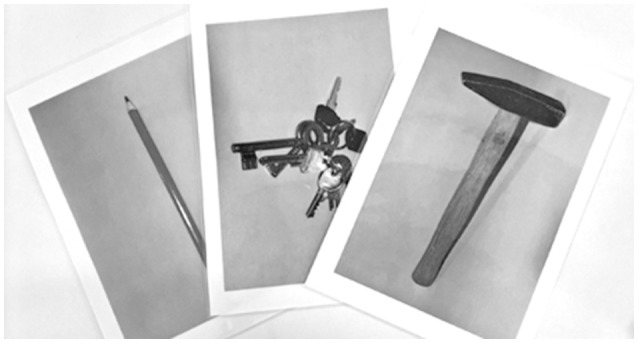
Pantomime of object use. Three of the 20 shown pictures.

Eleven patients diagnosed with dementia of the Alzheimer's typewith an age of 74.6a ± 7.6a (3 male, 8 female) and a Mini Mental State Examination score of 23.6 ± 2.9 (range: 18–28) and a control sample of 15 healthy, age-matched controls subjects (age: 71.5a ± 6.2a; 5 male, 10 female; **Table 2**) were assessed with the apraxia tests proposed by Goldenberg (15) (Figures 1–3), a reciprocal aiming task and an abstract sequencing task (reciprocal trail making task) proposed and described by Gulde (16) (see Table 1). The apraxia tests consisted of 10 imitations of meaningless finger gestures (max. score: 20), 10 imitations of meaningless hand gestures (max. score: 20), and 20 pantomimes (max. score: 55) (Figures 1–3). The reciprocal aiming task was to move with the dominant index finger a fast and accurate as possible between to marks (distance: 8.85 cm; 30 repetitions; outcome measure: frequency). The reciprocal trail making task was to point with the dominant index finger as fast and accurate as possible back and forth between a central mark and eight numbers from 03 to 24 (in steps of 3), which were set in a circular order (radius: 8.50 cm; outcome measure: trial duration).

**Table 1:** Overview of the applied tests and their outcomes.

**Test**	**Abbreviation**	**Outcome measure**	**Mean & standard deviation**
Imitation of meaningless finger gestures	Finger	Score based on correct imitations	15.91 ± 4.25 (6–20)
Imitation of meaningless hand gestures	Hand	Score based on correct imitations	18.55 ± 1.81 (15-20)
Pantomime of everyday actions	Panto	Score based on correct pantomimes	47.73 ± 7.71 (28-55)
Reciprocal aiming task	RAT	Frequency of target hits in Hertz	3.54 ± 1.24 Hz (1.95–5.51 Hz)
Abstract sequencing task (reciprocal trail making task)	RTMT	Trial duration in seconds	29.30 ± 16.21 s (10.07–54.52 s)

Six of the 11 patients and none of the control subjects revealed signs of apraxia on at least one of the three tests. Ethical approval was given by the local ethics committee (Faculty of Medicine of Technical University of Munich). All participants gave written informed consent.

A principal component analysis was run with the applied tests as variables. In the control sample age was added as a variable^1^. The number of components was preset to two. A prerequisite of the analysis was that the aiming task was measuring the latent component of a basal aspect of motor capacity and the trail making task was measuring the latent component of a basal aspect of cognitive capacity. For the rotation of the component matrix the varimax method was applied. The appropriateness of the principal component analysis was tested by the Kaiser-Meyer-Olkin criterion, sphericity by the Bartlett test, and the measures of sampling adequacy using anti-image matrices.

## Results

All results of the applied tests are listed in Table 2.

**Table 2:** Overview of the samples and their test results.

**Code**	**Age in[a]**	**Sex**	**Handedness**	**MMSE**	**Finger**	**Hand**	**Panto**	**RAT in [Hz]**	**RTMT in [s]**
P01	81	Male	Right	22	15^†^	19	55	2.82	11.98
P02	73	Male	Right	22	14^†^	15^†^	49	2.06	54.52
P03	76	Female	Right	22	19	18	53	2.86	22.88
P04	85	Male	Right	27	17	20	54	.243	10.07
P05	68	Female	Right	25	18	17^†^	47	5.27	38.73
P06	57	Female	Right	28	20	20	52	3.86	14.80
P07	70	Female	Right	24	18	20	52	4.67	10.99
P08	76	Female	Right	21	20	20	47	3.68	36.05
P09	80	Female	Left	26	17	20	41^†^	5.51	29.60
P10	78	Female	Right	24	11^†^	19	47	3.79	43.15
P11	76	Female	Right	18	6^†^	16^†^	28^†^	1.95	49.48
Patients (*n* = 11)	74.55 ± 7.57	3x male 8x female	10x right 1x left	23.55 ± 2.91	15.91 ± 4.25	18.55 ± 1.81	47.73 ± 7.71	3.54 ± 1.24	29.30 ± 16.21
Controls (*n* = 15)	71.47 ± 6.23	5x male 10x female	15x right 0x left	–	19.13 ± 1.19	19.40 ± 0.74	53.47 ± 1.60	3.12 ± 1.14	13.48 ± 3.03
Significance	*p* = 0.28	*p* = 0.74	*p* = 0.23	–	*p* = 0.03	*p* = 0.16	*p* = 0.03	*p* = 0.39	*p* = 0.01

*Keep in mind that the RTMT values for the control group are controlled for their motor capacity*.

† *Considered apraxic*.

### Patient sample

The Kaiser-Meyer-Olkin criterion was acceptable with 0.63. The test on sphericity was significant with a *p*-value of 0.02. The resulting communalities were all higher than 0.73 (Table 3), but the measures of sampling adequacy were in one case below 0.5 (Table 3). The two-component preset was confirmed by the Kaiser criterion (eigenvalue >1.0). The cumulated explained variance by the model was 80.5%.

**Table 3:** Patient sample: the outcomes of the principal component analysis.

**Variable**	**Communality (extraction)**	**Anti-image-correlation**	**“Cognitive” component^†^**	**“Motor” component^†^**
Finger	0.740	0.667	0.645	0.559
Hand	0.729	0.655	0.737	0.445
Panto	0.829	0.607	0.908	–
RAT	0.934	0.462^*^	–	0.966
RTMT	0.795	0.658	−0.875	–

* *Inappropriate (< 0.5)*,

† *Derived from the rotated component matrix, absolute values below 0.2 were excluded*.

### Control sample

The Kaiser-Meyer-Olkin criterion was acceptable with 0.57. The test on sphericity was non-significant with a *p*-value of 0.21. The resulting communalities were all, except Hand with 0.40, higher than 0.63 (Table 4) and the measures of sampling adequacy were in three of six cases below 0.5 (Table 4). The two-component preset was confirmed by the Kaiser criterion (eigenvalue >1.0). The cumulated explained variance by the model was 64.6%.

**Table 4:** Control sample: the outcomes of the principal component analysis.

**Variable**	**Communality (extraction)**	**Anti-image-correlation**	**“Cognitive” component^†^**	**“Motor” component^†^**
Finger	0.689	0.567	–	0.830
Hand	0.404	0.475^*^	0.474	0.432
Panto	0.627	0.497^*^	0.791	–
RAT	0.673	0.642	–	0.819
RTMT	0.724	0.448^*^	−0.849	–
Age	0.759	0.679	–	−0.865

* *Inappropriate (< 0.5)*,

† *Derived from the rotated component matrix, absolute values below 0.2 were excluded*.

**Figure 4:**
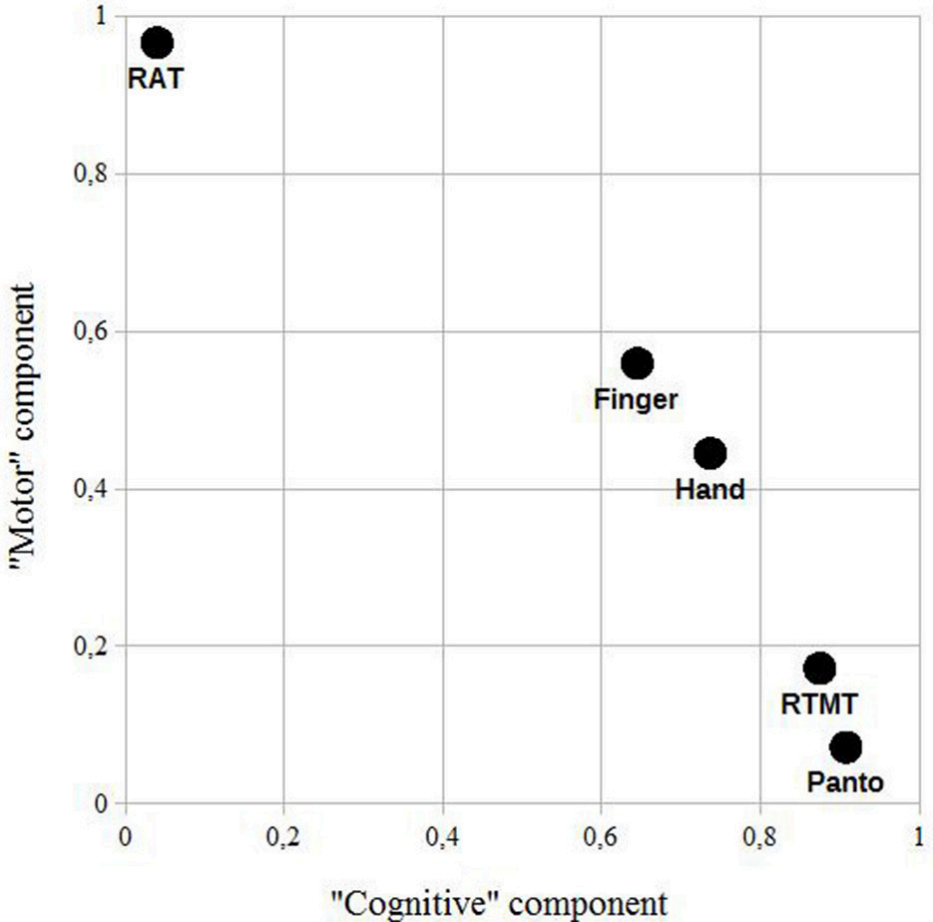
Patient sample: a rotated component plot using the absolute values of the rotated component matrix for a better visual inspection. RAT, reciprocal aiming task; RTMT, reciprocal trail making task; Finger, imitation of finger constellations; Hand, imitation of meaningless gestures; Panto, pantomime of the use of everyday objects.

**Figure 5:**
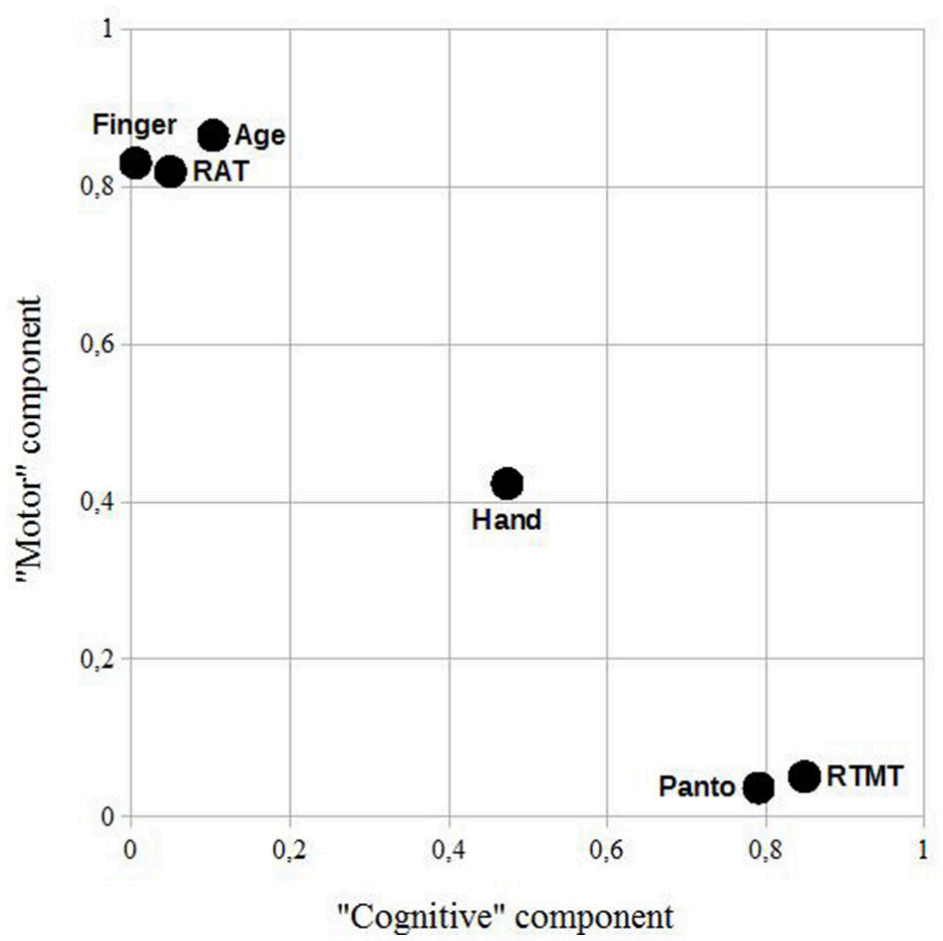
Control sample: a rotated component plot using the absolute values of the rotated component matrix for a better visual inspection. RAT, reciprocal aiming task; RTMT, reciprocal trail making task; Finger, imitation of finger constellations; Hand, imitation of meaningless gestures; Panto, pantomime of the use of everyday objects.

Figures 4, 5 illustrate the loading of the variables in the rotated component matrices, using the absolute values for a better visual inspection. The two latent components are named “motor” component and “cognitive” component as stated in the methods section.

## Conclusion

In this study we examined the scores of three apraxia tests: hand gestures, finger gestures, and pantomime (15). We further applied a test on maximum motor capacity and an abstract sequencing task (16), thought to be predominantly limited by its cognitive demands. The computed principal component analysis revealed the possibility of two latent components in the applied tests in both samples: the “motor” component and the “cognitive” component (please see **Limitations**). The factor loadings of Finger and Hand, the imitation of finger gestures, both revealed a strong motor component. A similar approach was used by Hartmann et al. (17) in stroke patients, examining the relationship of different activities of daily living and problem solving tasks applying multi-dimensional scaling (ALSCAL, SPSS). The present results, taken with care due to the clear limitations of this preliminary examination, indicate that the motor capacity can strongly influence the results of these tests, thought to assess impairments in higher order motor control (3) or as stated in 2014 by Goldenberg (18) “[…] the cognitive side of motor control”. Whether the strong influence of the motor component on Finger and Hand in comparison to Panto is based on specific neurodegenerative processes of dementia of the Alzheimer's type or is based on other factors than the motor capacity, which could be motivation or comorbidities like a beginning arthritis (one of the patients was diagnosed with arthritis), is questionable. In case of apraxia moderating the association of RAT and apraxia tests, Panto should as well be impacted and the RAT performance should differ between patients with and without signs of apraxia. However, a *post-hoc t*-test for independent samples revealed no significant difference (*p* = 0.93). If motivation plays a major role in the task performance, it would be difficult to explain why both imitations and pantomime tasks show such different degrees of impact, within and between the samples. The same accounts for possible arthritis, since Panto includes a lot of wrist and finger movements, especially fast rotations of the wrist. Concerning specific neurodegenerative processes, Mühlau et al. (19) did not find different neural representations other than in the motor cortex when comparing hand and finger gesture imitations in an fMRI study, while a lesion mapping study by Goldenberg et al. (20) revealed differences, concluding that a main difference between the tasks lies in the amount and types of visual information that needs to be processed (more in the hand gesture imitation). Interestingly, Finger and Hand were quite similar in the patient sample, while they clearly differed concerning the “cognitive” component in the control sample. Taking that a part of the RTMT is visually searching digits, the cognitive component could also be a visual component—but only in the control sample. This could be caused by healthy controls realizing the linear increment of the values in the RTMT [searching a defined value while patients would be exploring, see (16)]. Nevertheless, the strong association of Hand and Finger and RAT supports the dependence of both imitation tasks on motor capacity. In the control sample, the loading of Finger on the “cognitive” component is almost at zero, so it could be actually the case that the “cognitive” component is “cognitive” in the patient sample and “visual” in the control sample. This is further supported by the weak loading of age on this component, if it was “cognitive” in the control sample, age would be assumed to have shown an impact.

Concluding, taking into account that patient populations like dementia patients reveal motor impairments (11, 13, 21) (that can also be dependent on the type of dementia), the strong impact of motor capacity can influence the outcomes of such tests. We therefore suggest, apart from a similar analysis assessing larger samples, to control future tests on signs of apraxia by the assessment of basic motor capacity, e.g., by a reciprocal aiming task. The test assesses a basic aspect of motor capacity including speed-accuracy trade-off. It seems feasible that other tasks such as (sequential) finger tapping are also sensitive to reveal the motor component, this should however also be investigated in future experiments.

## Limitations

This pilot study has some limitations. A set of five variables was used to define two components. Further, the sample sizes (*n* = 11 and 15) were small and two patients were only able to perform the task according to its rules on a second attempt. However, the Kaiser-Meyer-Olkin criterion was acceptable [according to Kaiser (22) acceptable, but estimated “mediocre” in the patient sample and “miserable” in the control sample) with 0.63 and 0.57, respectively. In the control sample, the Bartlett test on sphericity was non-significant. This was due to controlling for maximum motor capacity in the RTMT in the control sample (Bartlett: *p* = 0.03 -> *p* = 0.21). Further, all communalities were above 0.73 in the patient and except one above 0.63 in the control sample, but the measure of sampling adequacy were in a total of four of 11 cases below a threshold of 0.5. For future studies we suggest sample sizes of at least 100 (23), although, as stated before, the communalities were high enough in this examination to give first insights into potential underlying components.

## Author contributions

PG, AA, and JH designed the study. SK, TG and JD-S diagnosed and selected the patients. KL and SK organized the patients' appointments and their transport. PG, KL, and AA performed the lab testing. PG performed the statistical analyses. KL, PG, and AA scored the apraxia tests. All authors contributed to the coordination of the study and the final draft of the manuscript.

### Conflict of interest statement

The authors declare that the research was conducted in the absence of any commercial or financial relationships that could be construed as a potential conflict of interest.

## Footnotes

^1^Age related effects in the patient sample were not considered, since it would have further led to including disease onset and progression, which are not quantifiable in a satisfactory way. Further, RTMT was controlled for the maximum motor capacity in the control sample by a regression leading to $RTMT^{{}^{\prime }}=RTMT+{\left(RAT-RAT_{\emptyset control}\right)}^{*}2.624$, since healthy elderly show dependence of RTMT on the RAT performance (16).

## Abbreviations

AD: Alzheimer's disease
Hand: Score in the meaningless hand gesture task
Finger: Score in the meaningless finger gesture task
Panto: Score in the pantomime of everyday actions task
RAT: Reciprocal aiming task
RTMT: Reciprocal trail making task.
